# Common Gene Therapy Viral Vectors Do Not Efficiently Penetrate Sputum from Cystic Fibrosis Patients

**DOI:** 10.1371/journal.pone.0019919

**Published:** 2011-05-27

**Authors:** Kaoru Hida, Samuel K. Lai, Jung Soo Suk, Sang Y. Won, Michael P. Boyle, Justin Hanes

**Affiliations:** 1 Department of Ophthalmology, The Johns Hopkins University School of Medicine, Baltimore, Maryland, United States of America; 2 Department of Biomedical Engineering, The Johns Hopkins University School of Medicine, Baltimore, Maryland, United States of America; 3 Department of Chemical and Biomolecular Engineering, The Johns Hopkins University, Baltimore, Maryland, United States of America; 4 Institute for NanoBioTechnology, The Johns Hopkins University, Baltimore, Maryland, United States of America; 5 The Center for Nanomedicine, The Johns Hopkins University School of Medicine, Baltimore, Maryland, United States of America; 6 Division of Pulmonary and Critical Care Medicine, Johns Hopkins Adult Cystic Fibrosis Program, The Johns Hopkins University School of Medicine, Baltimore, Maryland, United States of America; Ludwig-Maximilians-Universität München, Germany

## Abstract

Norwalk virus and human papilloma virus, two viruses that infect humans at mucosal surfaces, have been found capable of rapidly penetrating human mucus secretions. Viral vectors for gene therapy of Cystic Fibrosis (CF) must similarly penetrate purulent lung airway mucus (sputum) to deliver DNA to airway epithelial cells. However, surprisingly little is known about the rates at which gene delivery vehicles penetrate sputum, including viral vectors used in clinical trials for CF gene therapy. We find that sputum spontaneously expectorated by CF patients efficiently traps two viral vectors commonly used in CF gene therapy trials, adenovirus (d∼80 nm) and adeno-associated virus (AAV serotype 5; d∼20 nm), leading to average effective diffusivities that are ∼3,000-fold and 12,000-fold slower than their theoretical speeds in water, respectively. Both viral vectors are slowed by adhesion, as engineered muco-inert nanoparticles with diameters as large as 200 nm penetrate the same sputum samples at rates only ∼40-fold reduced compared to in pure water. A limited fraction of AAV exhibit sufficiently fast mobility to penetrate physiologically thick sputum layers, likely because of the lower viscous drag and smaller surface area for adhesion to sputum constituents. Nevertheless, poor penetration of CF sputum is likely a major contributor to the ineffectiveness of viral vector based gene therapy in the lungs of CF patients observed to date.

## Introduction

Nearly 20 years of clinical and laboratory research has thus far failed to realize successful gene therapy for cystic fibrosis (CF) [1], [2]. Viral gene vectors have dominated gene therapy efforts for CF, with adenovirus (AdV) and adeno-associated virus (AAV) representing two of the most widely tested systems to date [1], [2]. Although significant gene transfer has been observed for AdV and AAV vectors in cell lines and in a variety of animal models [3]–[5], neither vector has provided sufficient therapeutic end points in CF patients. Poor gene transfer has been attributed primarily to limited cellular uptake across the apical membrane of the lung airways, unproductive intracellular trafficking, vector toxicity, and immunological barriers [6].

We reasoned the CF sputum is a largely overlooked bottleneck that may limit gene therapy directed to the airways of CF patients. CF sputum possesses a bulk viscosity as high as 10^4^–10^5^ -fold greater than that of water at low shear [7], [8], and, thus, may physically exclude gene vectors from reaching epithelial cells. Although some viruses that infect mucosal surfaces (e.g. human papilloma virus and Norwalk virus) diffuse through human ovulatory cervical mucus as fast as through water [9], we recently found that other viruses (e.g. HIV and Herpes Simplex Virus) can be extensively trapped in non-ovulatory human cervicovaginal mucus collected from donors with healthy vaginal flora [10], [11]. Little is known about the ability of viral gene carriers commonly used in humans to penetrate CF sputum.

To investigate whether CF airway sputum serves as a transport barrier to viral vectors most commonly used in clinical trials, we performed high resolution multiple particle tracking (MPT) [12], [13] on both AdV and AAV serotype 5 (AAV5) in fresh, undiluted purulent sputum expectorated by CF patients. Although the average mesh spacing in purulent CF sputum (d ∼ 140±50 nm; range 60–300 nm) is substantially smaller than that in human cervicovaginal mucus (d ∼340±70 nm; range 50–1800 nm), the openings are sufficiently large that both AdV (d ∼80 nm) and AAV5 (d ∼20 nm) should readily penetrate CF sputum if they are not slowed by adhesive interactions with sputum constituents [11], [14].

## Results and Discussion

**Figure 1:**
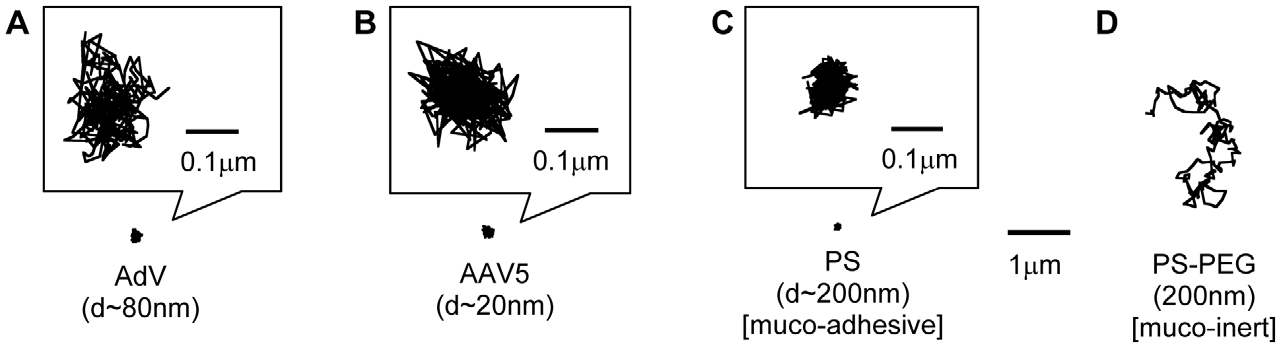
Sample 20 s trajectories. Representative trajectories of (A) Adenovirus (Adv), (B) AAV5, (C) muco-adhesive PS control nanoparticles and (D) muco-inert control PS-PEG nanoparticles. All trajectories have MSD values within one standard deviation of the ensemble average.

**Figure 2:**
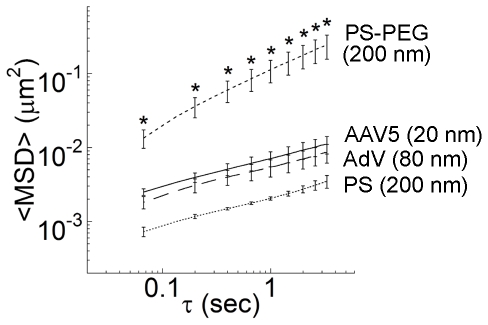
Averaged ensemble mean squared displacements <MSD> of viral vectors and synthetic particles with respect to time scale. Data represent n = 5 independent experiments with ∼100 particles tracked per experimental condition. Error bars represents standard error. *Statistically significant difference when compared with AAV5, AdV or PS (p<0.05).

Despite their native tropism for infecting airway cells, the diffusion of both AdV and AAV5 was strongly hindered in five of five independent human sputum samples. The highly constrained virus trajectories (Figure 1A & 1B) were similar to uncoated 200 nm polystyrene (PS) beads that are strongly immobilized in sputum (Figure 1C). We also tested 200 nm PS beads that were densely coated with low MW polyethylene glycol (PS-PEG), which we previously found to exhibit minimal adhesion to human mucus and CF sputum [11], [12], [14]. The PS-PEG particles exhibited traces spanning distances far larger than their diameters over the same duration in the same sputum samples (Figure 1D). We quantified the translational motions of particles and viruses by their time-scale dependent ensemble mean squared displacements (<MSD>). The ensemble-averaged <MSD> for AdV, AAV5 and muco-adhesive PS beads were all smaller than 10^−2^ µm^2^ across the time scales measured, whereas the <MSD> value for PS-PEG was at least 20-fold greater at a time scale of 1 s (Figure 2). AdV and AAV5 were slowed by over 3,000 and 12,000-fold in sputum compared to their theoretical speeds in water, respectively. In contrast, 200 nm PS-PEG particles were only slowed 40-fold in sputum as compared to in water. By fitting MSD vs. time scale (τ) to the equation MSD  = 4D_0_τ^α^, where D_0_ is the time scale-independent diffusion coefficient, an average value for α can be obtained that provides insight into the extent of impediment to particle motion (α = 1 for purely Brownian, unobstructed diffusion; the lower the value of α, the more constrained the particle motion). The average α-value of AdV and AAV5 (∼0.37 for each) is comparable to that for PS beads (∼0.39) and indicative of substantial impediment to free diffusion. In contrast, the average α-value for PS-PEG particles was ∼0.70, reflecting motions markedly less hindered by sputum across the time scales examined. The diffusive nature and substantially faster speeds achieved with muco-inert synthetic particles (d∼200 nm; roughly 2.5 times larger than AdV and nearly 10 times larger than AAV5), and the markedly larger average pore size for CF sputum compared to the size of both viral vectors, suggest that the hindered diffusion of adenovirus and AAV5 cannot be attributed to steric hindrance from a dense sputum mesh or to the high bulk viscoelasticity of CF sputum. Instead, both viral vectors are likely trapped in sputum by adhesion, and the small and hindered motions for AdV and AAV5 likely reflects, in part, thermal motions of mucin fibers to which the viruses are bound.

**Figure 3:**
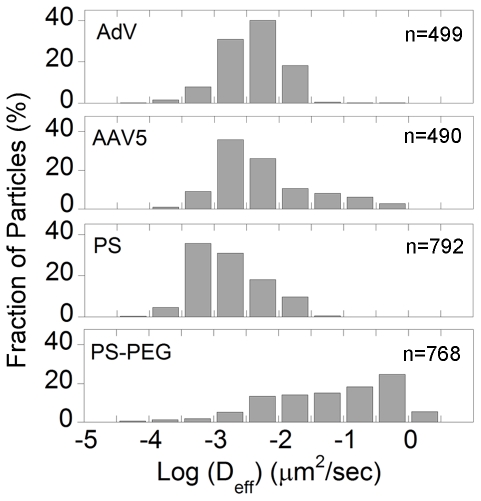
Distribution of logarithms of individual particle effective diffusivities (D_eff_). Distributions of D_eff_ of AdV, AAV5, PS and PS-PEG particles represented as a percentage of particles in CF sputum at a time scale of 1 s.

**Figure 4:**
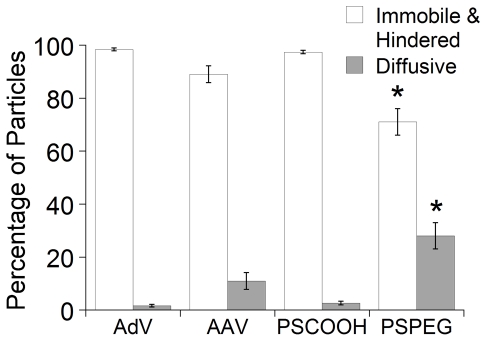
Transport mode distributions of AdV, AAV5, PS and PS-PEG particles. Particles were classified into either (i) immobile or hindered and (ii) diffusive [12], [15]–[17]. *Statistically significant difference when compared with AAV5, AdV or PS within the same transport mode classification (p<0.05).

Despite the slow ensemble averaged virus penetration speeds, there may exist fast moving ‘outlier’ virions within the sample capable of rapidly penetrating the sputum layer and, thus, mediating gene transfer. To evaluate the effectiveness of the sputum barrier, it is thus essential to measure the transport rates of all individual viral particles and, in particular, the speeds of the most rapidly moving fractions. The use of MPT allowed collection of quantitative data on the transport of individual particles, data that would otherwise be unavailable with ensemble methods such as FRAP. We plotted the distribution of the logarithms of individual particle effective diffusivities (D_eff_) at a time scale of 1 s (Figure 3). Over 30% of the 200 nm PS-PEG particles exhibited D_eff_ values greater than 0.1 µm/sec^2^, whereas only 0.4% and 0% of AdV and PS particles, respectively, exhibited such speeds (Figure 3). AAV5 had a higher fraction of faster moving particles, with 9% of virions displaying speeds greater than 0.1 µm/sec^2^. We used a Monte Carlo method that sorts the transport modes of particles based on their time-scale independent effective diffusion coefficients [12], [15]–[17], and found that over 98% of AdV and 90% of AAV were classified as either hindered or immobile, in good agreement with the distribution of effective diffusivities (Figure 4). It is not apparent whether the fast moving diffusive population of AAV5 represents a phenotypically unique subset of virions due to inherent vector packaging differences between individual virions, as the ratio of genomic particles to infective particles of AAV preparations can be as high as 200–10,000 [18]. It is unlikely that the differences in viral mobility can be attributed to the heterogeneity of the sputum microstructure, since the smallest pores present in CF sputum (range: 60–300 nm [14]) are substantially larger than the size of an AAV.

**Figure 5:**
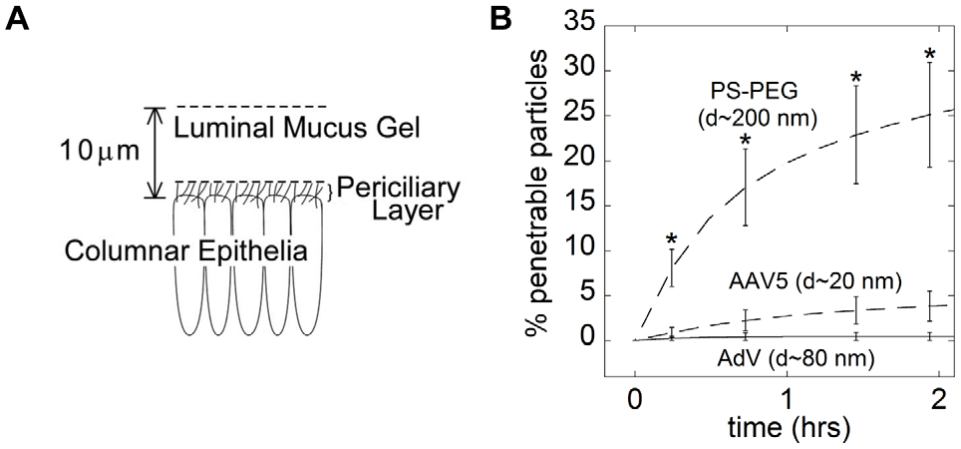
Theoretical model of particle penetration across a sputum layer. (A) *S*chematic of the model, where particles are deposited in airway lumen on top of the CF sputum layer and must penetrate a 10 µm sputum layer to reach the epithelial cells. The pericilliary layer is much smaller in CF patients due to the collapsed cilia from the accumulated sputum [60]. (B) Estimated fraction of viral and synthetic particles that are capable of penetrating a 10 µm thick layer of CF sputum over time using Fick's second law. *Statistically significant difference when compared with AAV5, AdV or PS (p<0.05).

Based on our MSD measurements at short time scales, we used Fick's second law to estimate the fraction of AdV and AAV5 that may penetrate a sputum layer of a given thickness over time (Figure 5A). Although the thickness of CF sputum coating airways epithelial cells varies from patient to patient, by location within the respiratory tract, and likely depends on disease progression, most estimates are in the range of 10–55 µm [19]–[21]. Assuming the sputum layer to be 10 µm thick, only 0.4 and 2.7% of AdV and AAV5 are expected to penetrate after one hour, respectively (Figure 5B). In contrast, roughly 20% of the PS-PEG particles may penetrate the sputum layer over the same duration. If the sputum layer were 55 µm thick, we predict only 0.2% of AdV and 0.7% of AAV5 would be capable of penetrating within one hour, as compared to nearly 40% of PS-PEG particles.

While this analysis provides insight into the extent sputum may serve as a barrier to viral vectors, the fraction of vectors that can mediate gene transfer will likely be much smaller than this estimate. Upon reaching the epithelial cells, the glycocalyx and the paucity of receptors on the apical membrane further limit cell entry [6], [22]. The efficiency of AAV entry into unpolarized cells was 13% [23] and likely even lower for polarized epithelium [24]. After endocytosis, numerous potentially rate-limiting intracellular events must also be overcome for successful transduction [25], [26]. Nevertheless, the limited gene carrier penetration observed here in purulent CF sputum helps to explain the very poor gene transfer observed with AAV in the human airways as compared to human nares, since CF sputum in the lung likely constitutes a far more tenacious barrier than sputum in human nares or mucus in the lungs of other animals [27], [28]. Previously, aerosol doses as high as 2.5×10^10^ infectious adenovirus (AdV) were administered to CF patients, but ≤2.4% of airway cells were shown to contain nuclear localized vector DNA, as estimated by fluorescence in situ hybridization experiments [29]. A major Phase II clinical trial utilizing AAV was also recently dropped due to inadequate efficacy, even though efficient gene transfer was evident in both rabbits and monkeys [30], as well as in the maxillary sinuses of humans [31].

The extracellular fluids in the lung have long been considered an integral part of a complex barrier that minimizes infections of the airway epithelia. For example, the airway surface liquid has both antimicrobial and antiviral properties, with high concentrations of lysozyme [32] and antibodies [33]. Antibodies were found to limit adenoviral infection of airway epithelium in both animal models and humans [33]–[35]. In CF patients, proteins associated with chronic inflammation, such as high concentrations of uninhibited neutrophil elastase [36], may further decrease vector efficiency. Recent studies have shown that bronchoalveolar lavage [29] fluid from CF patients reduces both AdV and AAV2-mediated gene transfer [37]–[39]. Although it has been reported that AAV5 transduction is maintained in the presence of BAL fluids from CF patients [40], 20% of the population harbor neutralizing antibodies against AAV5 [41]. The results presented here indicate that CF sputum can also act as a critical diffusional barrier, trapping viral vectors via adhesion and, thereby, limiting penetration. The reduced penetration speeds of viruses in sputum are also likely to increase the probability of viral inactivation by native protective mechanisms.

Although the specific nature of the adhesive interactions between viral vectors and sputum constituents remain unclear, it is likely a complex interplay of adhesion based on hydrophobic, ionic, hydrogen-bonding and/or van der Waals interactions. Mucins, the primary building block of CF sputum, are highly flexible molecules that are densely glycosylated, and therefore carry a negative charge due to the presence of carboxyl or sulfate groups. In addition, they also contain periodic hydrophobic “naked” globular regions interspersed along the mucin fibers, stabilized by multiple internal disulfide bonds [42]. Thus, mucins may form hydrophobic, electrostatic and/or hydrogen bonding adhesive interactions with foreign particles [43]. Elevated levels of bacterial and endogenous DNA, as well as actin filaments from degraded neutrophils in CF sputum, further contributes to its dense mesh structure and increased adhesivity [43]–[45]. The high density of adhesive domains, coupled with the highly flexible nature of mucins and other macromolecules, allows formation of multiple adhesive interactions with surfaces of foreign particles [43]. Even if each adhesive interaction is low affinity and can be readily disrupted by thermal energy, a large number of low-affinity adhesive interactions with the sputum mesh can effectively immobilize particles with permanent high viscidity [46]. Alternatively, it is possible that some viruses may bind directly to specific domains along mucins. For example, AAV4 binds to O-linked sialic acids highly expressed on mucins in CF sputum, and presence of MUC1 inhibited gene transfer with AAV4 [47]. While AAV5 also binds to 2,3-linked sialic acids, AAV5 interacts with sialic acids on N-linked carbohydrates which are rarely expressed on mucins, and MUC1 did not block gene transfer with AAV5 [47].

Although a number of scientists initially speculated that only 6–10% of airway epithelial cells need to have CFTR corrected to restore Cl^−^ transport in an in vitro cell culture model [48], it is now thought that a larger number of cells must be transduced for the normalization of both Cl^−^ secretion and Na^+^ absorption [49]. While the number of cells that need to be genetically corrected for clinical benefit remains unresolved, improved gene carrier penetration across the sputum layer is likely a critical step towards more reliable gene transfer in the CF lung airways. Our results suggest that engineering muco-inert surfaces on virus-sized particles can greatly improve vector penetration through purulent CF sputum. One possible approach may be to engineer viral vectors lacking specific surface epitopes that cause them to adhere to sputum constituents. For example, Muzyczka and colleagues have engineered AAV2 mutants without the normal heparin sulfate binding of the virus and discovered that the mutant virus produced a larger area of transduction upon injection in the striata of rat brains (unpublished observations; personal communication). This was presumably due to the increased diffusion of mutant AAV2 in the brain. In the scenario where specific epitopes responsible for mucoadhesion is not known, an alternative approach may be to employ directed evolution techniques to evolve non-mucoadhesive viruses. This approach is exemplified by previous work that evolved AAV vectors more efficient at CFTR delivery to human ciliated airway epithelium, or AAV vectors that evade neutralizing antibodies [50], [51].

Alternatively, vector penetration across sputum may be enhanced with adjuvant therapies targeted at reducing the barrier properties of CF sputum, such as bronchoalveoloar lavage (BAL) to reduce sputum, or clinically prescribed mucolytics that degrade specific constituents of the sputum mesh [43]. Two commonly prescribed mucolytic agents, N-acetyl-cysteine (NAC; Mucomyst ®) and recombinant human DNAse (rhDNAse; Pulmozyme ®), have been shown to reduce the bulk rheological properties of CF sputum [52]–[54]. Dawson *et al* previously found that rhDNase treatment failed to improve the diffusion of polystyrene nanoparticles in CF sputum [7], whereas Suk *et al* showed that NAC markedly enhanced the diffusion of coated, muco-inert nanoparticles in CF sputum, but not the diffusion of uncoated particles that were muco-adhesive [55]. It remains to be determined whether these mucolytic treatments will enhance or hinder viral vector transport.

We have demonstrated that human CF sputum is likely a critical barrier to overcome for successful CF gene therapy. Much of the current effort on improving AAV vectors for CF gene therapy has focused on identifying and characterizing novel variants of AAV with improved lung tropism. For example, AAV serotype 1 and 6 has been shown to transduce airway epithelia in mice or polarized human cell cultures significantly more efficiently than the most widely tested serotype, AAV2 [56], [57]. Further efforts have led to the development of AAV6.2, with a single point mutation and a 2-fold improvement in the transduction of airway epithelium compared to parental AAV6 vectors in both in vivo mouse studies and in vitro human ciliated airway epithelium [58]. However, improved vector penetration across sputum remains a critical step to reduce the viral dosage necessary for efficient transduction in the CF lung. Our work further suggests that high transgene expression in cell culture models or in animal models may not be sufficient justification to initiate clinical trials in CF patients without evidence that the same systems are capable of penetrating sputum.

## Materials and Methods

### Ethics Statement

Sputum samples were collected at the Johns Hopkins Adult Cystic Fibrosis Program conforming to ethical standards of the Johns Hopkins Medicine Institutional Review Board. Written informed consent was obtained from all participants.

### Collection of Cystic Fibrosis Sputum

Same day samples were placed on ice upon collection, pooled together to minimize patient-to-patient variability and used within 24 hrs. The barrier properties of collected sputum were confirmed by tracking fluorescent polystyrene (PS) beads that undergo polyvalent adhesive interactions with sputum [14].

### Preparation of Fluorescent Viruses and Particles

Green fluorescent protein–labeled AdV was constructed and generously provided by Dr. Curiel (University of Alabama). AlexaFluor 488 labeled AAV5 was provided by Dr. Chiorini (NIH). Fluorescent uncoated polystyrene nanoparticles with carboxyl-modified surfaces (Molecular Probes, Eugene, OR) were used as provided. PEG-coated nanoparticles were prepared by covalently modified surface carboxyl groups with polyethylene glycol (PEG) as described previously [12], [14]. Size and ζ-potential (surface charge) of nanoparticles were determined by Zetasizer Nano ZS90 (Malvern Instruments, Southborough, MA) to confirm dense PEG conjugation [12], [59].

### Multiple Particle Tracking (MPT)

Fluorescently labeled virions were added to ∼30 µl of CF sputum at minimal dilution (3% v/v), placed in a custom made glass chamber and incubated for 30 minutes prior to microscopy. Movies were captured on an EMCCD camera (Cascade II: 512, Photometrics, Tucson AZ) mounted on an inverted epifluorescence microscope (3-I Marianas, Zeiss, Thornwood, NY) equipped with a 100X oil-immersion objective (numerical aperture 1.3). Movies were recorded at a temporal resolution of 66.7 ms for 20 s using Slidebook 4.2 Advanced Imaging software (Universal Imaging Corp. Downington, PA). Movies were analyzed with Metamorph software (Universal Imaging Corp. Downington, PA) to extract x, y positional data over time. Time-averaged mean square displacement (MSD) and effective diffusivity.

(D_eff_) for each particle were calculated as a function of time scale (s) [12], [14]. CF sputum was assumed to be locally isotropic but not necessarily homogeneous; thus, 2D diffusivity is equal to 3D diffusivity (see review [13] for more details). Five independent experiments in sputum from different days, with n∼100 virions per experiment, were performed. Average transport rates were calculated by geometric ensemble-averaging of individual transport rates. Particle transport mechanism (immobile, hindered, and diffusive) was classified as discussed previously [12], [16]. Briefly, the mechanism of particle transport over short and long time scales was classified based on the concept of relative change (RC) of D_eff_. RC values of particles at short and long time scales were calculated by dividing the D_eff_ of a particle at a probed time scale by the D_eff_ at an earlier reference time scale. By calculating RC values for two time regimes (i.e., short and long time scales), one can obtain the transport mode that describes the particle transport properties over different length and temporal scales. Particles undergoing hindered diffusion are expected to possess RC values below that for typical diffusive particles. Due to the random nature of diffusion, however, the RC values of a population of purely diffusive particles will have a certain spread around the value 1. Therefore, a Monte Carlo simulation of 10,000 random walks was used to predict this distribution of RC values (please see [16] for more details). The tracking resolution was 10 nm, as determined by tracking particles immobilized with a strong adhesive [12], [13].

### Particle Penetration Model

Speeds of individual particles, obtained from particle tracking data, were projected to 2 hours using the measured <MSD> versus τ relationship. Concentration profiles over time were obtained by numerically integrating Fick's second law, du/dt  =  D_eff_ d^2^u/dx^2^ with the initial condition of particles present at the apical side (u(x,0)  = 1 when x  = 0, u(x,0)  = 0 otherwise) and boundary conditions of constant particle concentration at the apical side (u(0,t)  = 1) and no flux out across the epithelium (du/dx|_x = 10_  = 0).

### Statistical Analysis

Statistical significance between two groups was determined with the one-sided student's t-test under the assumption of unequal variance. P-values less than 0.05 were considered significant.
